# Owning what one did not make: psychological ownership, agency, and the authorship legitimation deficit in human–AI co-creation

**DOI:** 10.3389/fpsyg.2026.1928186

**Published:** 2026-10-28

**Authors:** Shuying Hong, Qianqian Li, Ziran Zhou, Yanjiao Dong, Yueh-Chih Hsiao, Qi Cao

**Affiliations:** 1 Department of Art and Design, Guangzhou Institute of Science and Technology, Guangzhou, China; 2 Department of Visual Design, Institute of Design Research, Hanyang University, Ansan, Republic of Korea; 3 Department of Mathematics, Faculty of Science, National University of Singapore, Singapore, Singapore; 4 Department of Preschool Education, College of Education, Anyang Normal University, Anyang, China; 5 Department of Technology Application and Human Resource Development, National Taiwan Normal University, Taipei, Taiwan, China; 6 Department of East Asian Studies, Graduate School of International Studies, Hanyang University, Seoul, Republic of Korea; 7 School of Humanities and Social Science, The Hong Kong University of Science and Technology, Kowloon, Hong Kong SAR, China

**Keywords:** authorship, conceptual analysis, creative selfhood, generative artificial intelligence, human–AI co-creation, psychological ownership, responsibility attribution, sense of agency

## Abstract

Authorship can align three relations to a work: experiencing it as one’s own, feeling like the agent behind it, and standing answerable for it. Effortful engagement can support this alignment, an arrangement this article calls the labor nexus of authorship, but direction, selection, editing, and authority over release also matter. Generative artificial intelligence can separate initial-production effort from the resulting product without uniformly weakening ownership, agency, or responsibility. This conceptual analysis specifies an authorship legitimation deficit as a mismatch between a particular authorship designation and the experiential or contribution-based grounds expected to support it under prior domain criteria. Separate ownership, agency, and community-warrant profiles distinguish an unsupported sole-creator claim from a supported editorial role, even when the person’s contribution and experience are unchanged. Historical precedents locate the problem within delegated and distributed making. The main contribution is a role-relative conceptual classification. Conditional relational hypotheses concern refusal or qualification of authorship (P4) and later recognition of alternative contributions (P6); agency and observer credit-blame hypotheses (P5a/P5b) concern constituent concepts rather than effects of the deficit. Market dependence suppresses observable disavowal when qualification threatens income; perceived system agency conditions felt answerability while assigned release duties remain fixed. Contribution records may assist later recognition but do not guarantee it. The framework identifies when a role exceeds its grounds while preserving supported AI-assisted authorship and accountability for release.

## 1 Introduction

A person can feel little ownership of AI-generated text while continuing to declare themselves its author, a dissociation documented by Draxler et al. (2024) in personalized text generation. Consider a hypothetical designer who selects an AI-generated illustration for publication. The designer may regard the result as accomplished and marketable while questioning whether a sole-creator designation describes the contribution made. The issue is not merely private feeling. It concerns what people say about their work, how their contribution is presented, and where they locate responsibility for releasing it. A public designation can persist despite weak felt ownership; whether the person accepts, refuses, or qualifies that designation is a further question.

This article treats that divergence as a problem for psychology, and specifically for the psychology of how a person comes to experience an output as their own. Three relations can align in being an author. The first is psychological ownership, the felt state in which a target is experienced as “mine” (Pierce et al., 2001, 2003). The second is the sense of agency, the experience of being the author of one’s actions and, through them, of their effects in the world (Moore, 2016; Haggard, 2017). The third is responsibility, which includes distinct questions of attributability, answerability, and accountability (Shoemaker, 2011). In creative work, effortful engagement can support ownership by recruiting control, intimate knowledge, and self-investment (Pierce et al., 2003), and it can supply predictive and retrospective cues for agency (Synofzik et al., 2008; Moore and Obhi, 2012). Observers may use evidence of contribution to assign credit, while responsibility can depend on knowledge and authority over approval or release. We call their possible alignment the labor nexus of authorship, an arrangement in which making, owning, and being answerable can support one another. The term names a contingent configuration of familiar relations rather than a second new psychological construct.

This conceptual analysis concerns the conditions under which a person can experience an output as their own work, feel like the agent behind it, and stand answerable for it, and how those relations change when production is delegated. It situates the problem within theoretical and philosophical psychology by examining psychological states together with the standards used to recognize creative contributions. Legal rights, social recognition, and justified responsibility inform the context but do not substitute for measures of ownership or experienced agency. The question is how a specific authorship role can be supported when experience, contribution, and designation diverge.

Generative systems can unsettle this alignment by performing parts of production that a person would otherwise undertake. In workflows limited to specification of intent and selection among outputs, less human effort may be needed to realize the initial work. Other workflows retain substantial human direction, revision, and checking. Recent studies distinguish the resulting outcomes: in short-story writing, access to generative assistance raised evaluated creativity, with larger gains for less creative writers, while reducing collective diversity (Doshi and Hauser, 2024); in poetry writing, McGuire et al. (2024) compared editing an initial AI-generated poem with iterative co-creation and measured creativity and creative self-efficacy. The latter study does not establish recovery of psychological ownership. An editor may make substantive revisions even when the system supplied the initial draft. These findings motivate attention to the human role and the outcome measured rather than a general inference that a better product weakens its maker’s ownership.

Existing constructs capture different relations within this problem. Psychological ownership theory explains how control, knowledge, and self-investment can produce the feeling of mineness; the feeling alone does not determine whether a public authorship role appropriately recognizes a contribution. Research on the sense of agency informs the distinction between control over immediate outcomes and the distal, curatorial authorship exercised through prompting, selection, and revision (Limerick et al., 2014). Responsibility research raises a further distinction between a user’s experienced obligation and others’ judgments about that user. The aim is to clarify and reorganize the relations among authorship, ownership, agency, and responsibility under conditions of human–AI co-creation, and to specify when a designation exceeds its supporting grounds.

The contribution is a single construct and the model that surrounds it. An authorship legitimation deficit is a mismatch between a specified authorship designation and the experiential or contribution-based grounds expected to support it under prior domain criteria. Legitimation names the warrant for that particular role, not a universal requirement that every author perform the work’s physical execution. A community’s recognition of a claim and the normative justification of its criteria remain distinct questions. When the designation of authorship outruns its supporting grounds, a deficit opens. We first decompose the labor nexus into routes to ownership, agency, and externally assigned answerability (Figure 1). We then examine three outcome domains: observable authorial disavowal, in which a person refuses or qualifies an offered designation; changes in the user’s felt answerability and in observers’ credit and blame judgments, which may follow different directions; and a reorganization of legitimation, in which communities may recognize direction, selection, editing, or verification as grounds for particular roles. Responsibility is examined as an outcome rather than included in the deficit’s diagnostic indicators. Conditions governing these relations are specified separately.

**Figure 1:**
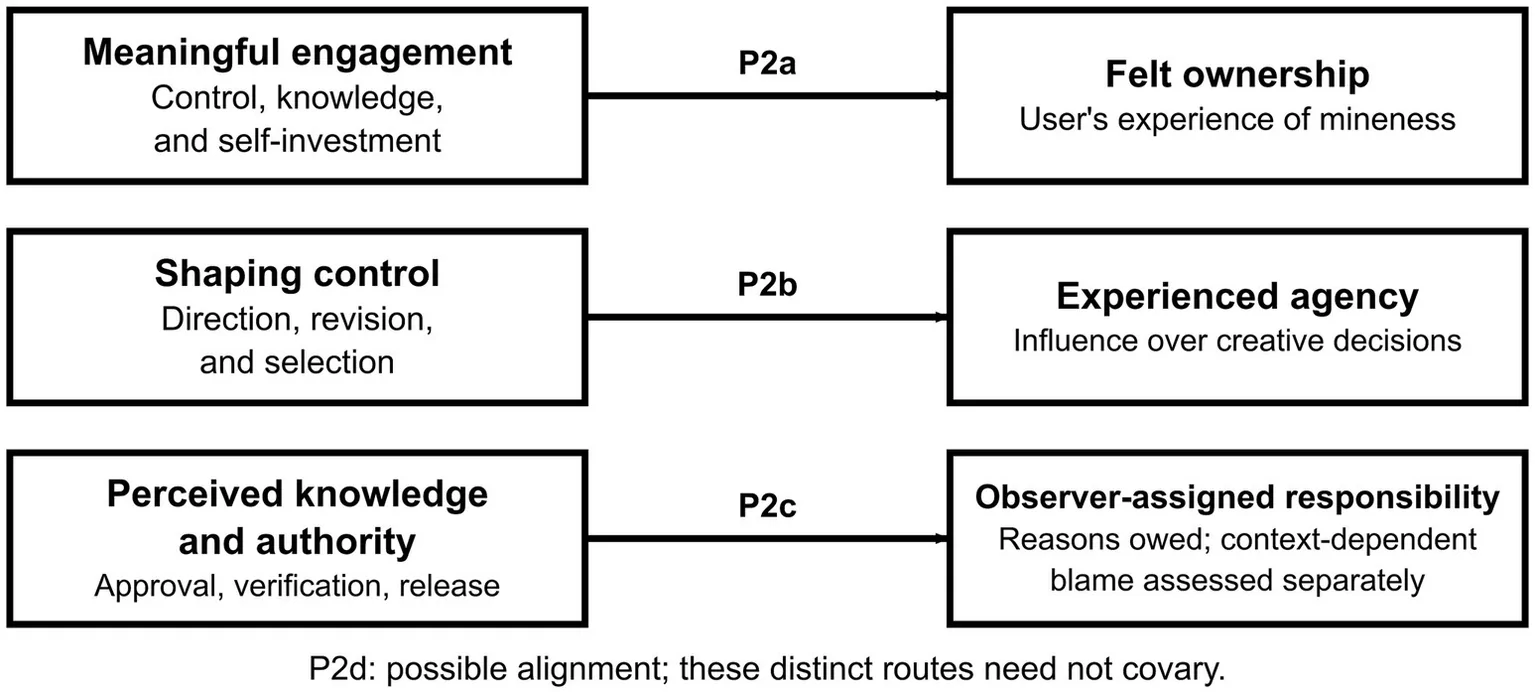
Separate routes within the labor nexus. Solid arrows show proposed empirical relations: meaningful engagement supports felt ownership (P2a), shaping control supports experienced agency (P2b), and perceived knowledge and approval or release authority support observers’ judgments of reasons owed and, under the relevant harm and avoidability conditions, blame (P2c). Responsibility judgments are assessed separately from creative credit and are not indicators of the authorship legitimation deficit. P2d denotes possible alignment among these distinct routes, an analytic description without an additional causal path, serial mediation, or required co-variation. P2 summarizes the workflow-specific ownership and agency predictions; Figure 2 separately represents deficit diagnosis.

The remainder of the article proceeds as follows. Section 2 reconstructs the labor nexus of authorship in psychological terms, distinguishing the routes through which engagement can support ownership, agency, and answerability. Section 3 examines generative production alongside earlier cases of delegated making and specifies the decoupling of initial-production effort from product. Section 4 explains the relation the proposed framework adds to the existing psychological concepts. Section 5 defines the authorship legitimation deficit and distinguishes it from adjacent notions of authorship and ownership. Section 6 states the propositions and mechanisms and examines the three outcome domains. Section 7 specifies boundary conditions and moderators. Section 8 draws implications for theory and future research, Section 9 discusses the limits of the account, and Section 10 concludes.

## 2 The labor nexus of authorship: making, owning, and being responsible

To see what the decoupling of effort and product may disturb, it is necessary to state the arrangement it acts upon. In some creative workflows, feeling that a work is one’s own, feeling like its agent, and being answerable for it are supported by related forms of engagement. We call this arrangement the labor nexus of authorship. Its elements are familiar in isolation; the point of naming the nexus is to examine how they can align at three levels. At the psychological level, making can recruit experiences of ownership and agency. At the behavioral level, effort is observable activity through which a person’s contribution can be enacted and made legible. At the normative level, particular contributions can furnish grounds for a recognized role and its responsibilities. The amount of effort alone does not secure this alignment: direction, execution, and authority over release may belong to different people. Table 1 summarizes the concepts discussed in this section and anticipates how each may be reorganized under generative AI.

**Table 1:** Key concepts and their reorganization under generative AI.

Concept	Meaning or supporting route	Possible divergence under generative AI	Distinction in the proposed framework
Authorship	Public assignment of a creative role under domain-specific criteria	Designation can persist while production effort or experience changes	Evaluate the specified role against its prior experiential or contribution grounds
Psychological ownership	Felt state of a target as “mine,” grown through control, intimate knowing, and investment of self	Delegation may reduce engagement; consequential prompting, selection or editing can recruit the routes	Felt ownership is distinct from a warranted contribution role
Sense of agency	Experience of authoring one’s actions and their effects, built from action–effect cues	Agency over selection and revision can differ from agency over initial production	Assess distal shaping decisions separately from immediate action-effect episodes
Responsibility attribution	Users’ felt obligation and observers’ reasons owed or blame are distinct judgments	Reduced experienced agency can coexist with retained release duties and observer blame	Responsibility outcomes are separate from deficit diagnosis and justified accountability
Creative selfhood	Sense of oneself as a creative person, which meaningful making can support	Delegation can change participation without removing direction or substantive editing	Creative identity is distinct from psychological ownership and creative self-efficacy
Legitimation	Warrant for a specified role under prior experiential or contribution criteria	A claimed role may exceed the grounds supplied by its workflow	Direction, selection, editing or verification can supply role grounds; recognition requires normative appraisal

### 2.1 Making and owning: effort as a route to psychological ownership

Psychological ownership is the state in which a person experiences a target, material or immaterial, as theirs, independently of any legal title (Pierce et al., 2003; Dawkins et al., 2017). It is not a metaphor for possession but a measurable feeling with identifiable antecedents. Pierce and colleagues specify three routes through which the feeling develops: exercising control over the target, coming to know it intimately, and investing the self in it (Pierce et al., 2001, 2003). Effortful making can recruit all three. Producing and revising a work can allow a person to control its coming-into-being, know it through the process of making, and invest time, skill, and self in it. The investment of self is not incidental. On the classic account of the extended self, things a person makes and holds can become part of how the self is constituted and recognized (Belk, 1988, 2013). Field research relates psychological ownership to employee attitudes and behavior (Van Dyne and Pierce, 2004), while territorial behavior can express and defend claims of ownership (Brown et al., 2005). These relations concern the character of engagement, not simply the duration of labor. Specifying, selecting, or editing can also recruit control, knowledge, and self-investment when those decisions meaningfully shape the work.

Labor-based valuation supplies a related but distinct comparison. The effort heuristic concerns the use of perceived effort to judge quality, value, and liking, particularly when quality is ambiguous (Kruger et al., 2004). The IKEA effect concerns increased valuation of self-made products, with successful completion as an important boundary (Norton et al., 2012). Neither valuation measure establishes psychological ownership or the legitimacy of an authorship role. Similarly, Brasel and Gips (2014) examine touch interfaces and ownership in product evaluation, not prompting versus making in creative tasks. Together these literatures justify examining engagement and effort while keeping ownership, valuation, and recognized contribution separate.

### 2.2 Making and agency: cues for the sense of authorship

The sense of agency is the experience of being the author of one’s actions and, through them, of their effects (Moore, 2016; Haggard, 2017). It is not a direct read-out of causal structure. It is constructed from cues, both the predictive signals that accompany intended action and the retrospective fit between what one intended and what occurred (Synofzik et al., 2008). People can feel in control when they are not, and can fail to feel in control when they occupy a central causal role (Moore, 2016). Effortful making can supply these cues in a rich form: the person forms an intention, acts to realize it through a sequence of steps, and experiences the emerging work through the coupling of intention, action, and result. Creative labor can thus offer repeated evidence that the work is responding to one’s decisions. Where effort involves little discretion, however, substantial labor can coexist with weak experienced agency. Conversely, selecting or revising generated material can preserve control over consequential decisions even when detailed production is delegated.

One well-studied signature of agency is the perceived compression of time between a voluntary action and its outcome (Moore and Obhi, 2012). Intentional binding concerns specific action-effect episodes; its validity as a measure of authorship over an extended text or image cannot be assumed. In a creative workflow, agency over selecting a candidate may differ from agency over its detailed production. The relevant decision and outcome must therefore be specified when assessing the distal, curatorial form of authorship.

### 2.3 Making and responsibility: felt and assigned answerability

Responsibility includes distinct questions about whose commitments an action expresses, who owes reasons, and who can be held accountable (Shoemaker, 2011). Experienced agency can inform a person’s sense of being answerable for an outcome (Haggard and Tsakiris, 2009; Moore, 2016), but that experience is not the sole ground of responsibility. Here, felt answerability concerns a user’s reported obligation to explain and stand behind an output; externally assigned responsibility concerns others’ judgments directed at that user. A person who selected, checked, approved, or published a work may owe reasons even if they feel little ownership of it. For observers, knowledge of relevant decisions and authority over approval or release provide grounds for identifying the answerable party. Where foreseeable harm and avoidable decisions are involved, they may also inform blame. The user’s experience and the observer’s judgment are therefore separate outcomes. Neither by itself establishes whether responsibility is normatively justified. Delegating production can alter the experience of making while leaving obligations attached to approval and release intact.

### 2.4 What the nexus secured, and its decomposition

Two qualifications sharpen this account. First, works are realized through the coordinated activity of many contributors rather than by solitary makers, so authorship was never a transparent reflection of individual making (Becker, 1982). Credit is attributed to a focal author by convention, even as the work depends on distributed labor. Second, recognition is allocated unequally and accrues disproportionately to those already recognized (Merton, 1968). These qualifications matter because they show that authorship is an attribution layered over a distributed process. Where the labor nexus aligns, it does not require that a work issue from a single pair of hands. Direction, interpretation, execution, and verification can supply different grounds for different roles. A focal designation can be supported without attributing every contribution to the named person, while the person who undertook the most labor can remain under-recognized.

Hypothetical manager-secretary and architect-contractor cases make the distinctions concrete. A manager can approve an email drafted by a secretary and retain responsibility for its release; the secretary supplied its composition. An architect can originate and direct a design without performing construction, while a carpenter contributes workmanship rather than originating the design. Each can be answerable for different decisions. These examples separate physical effort from creative direction and authority. They also show why felt ownership can belong to a contributor who lacks the focal byline, and why a supported low-execution-effort role need not involve an authorship deficit.

The nexus can be stated more precisely as a set of routes, which allows changes in its alignment to be located. Meaningful engagement is predicted to support psychological ownership through control over the work, intimate knowing of it, and investment of the self in it (P2a). Effective shaping control is predicted to support task-specific agency through the coupling of intention, action, and outcome (P2b). Perceived knowledge and authority over approval or release are predicted to support observers’ judgments of reasons owed and, where the relevant harm and avoidability conditions apply, blame (P2c). These routes can align in the labor nexus (P2d), an analytic description rather than a claim that the three must covary or form a serial chain. P2 summarizes the ownership and agency predictions when delegation reduces meaningful engagement or shaping control; responsibility grounded in release authority can persist. Figure 1 presents the three routes. Their possible alignment and the later diagnosis of a role-ground mismatch are kept distinct. These propositions are collected, alongside the model’s other propositions, in Table 2.

**Table 2:** Propositions and theoretical bases of the authorship legitimation deficit model.

Label	Relation	Proposed claim and status	Theoretical basis
P1	Delegation → initial-production effort	Empirical: delegation lowers initial effort at matched quality	Noy and Zhang (2023)
P2	Delegation → changed experiential routes	Empirical: loss of engagement or control weakens experience	Pierce et al. (2003); Synofzik et al. (2008)
P2a	Engagement → psychological ownership	Empirical: control, knowledge and self-investment support ownership	Pierce et al. (2003); Van Dyne and Pierce (2004)
P2b	Shaping control → experienced agency	Empirical: shaping control supports task-specific agency	Synofzik et al. (2008); Moore and Obhi (2012)
P2c	Release knowledge/authority → reasons owed and conditional blame	Empirical: knowledge/authority support reasons owed and scoped blame	Shoemaker (2011)
P2d	Possible alignment of the three routes	Analytic: the routes can align without required covariance	Present article
P3	Specified role relative to prior grounds	Analytic: inadequate applicable role grounds identify a mismatch	Present article
P4	Role-ground mismatch → refusal or qualified authorship	Relational hypothesis: mismatch predicts refusal or qualification under accepted criteria and low costs	Present article; Draxler et al. (2024), boundary evidence
P5a	Agency → felt answerability, duties fixed	Component hypothesis: agency predicts felt answerability at fixed release duties	Present article; Ciardo et al. (2020)
P5b	Matched AI-versus-human delegation contrasts	Comparative hypothesis: AI delegation reduces credit more than blame	Porsdam Mann et al. (2023)
P6	Recurrent mismatch → new recognized grounds	Relational hypothesis: recurring mismatch predicts alternative role uptake with assessable contributions	Present article; Beckert (2009); Epstein et al. (2023)
B1	Prior effort criterion informs P3	Criterion: valued effort can constrain low-execution claims	Kruger et al. (2004)
B2	Market dependence moderates P4	Moderator: threatened income suppresses refusal/qualification	Present article
B3	Prior origination criterion informs P3	Criterion: singular-origination norms constrain delegation	Becker (1982)
B4	Baseline records condition P6	Condition: prior records support later recognition	Present article; Beckert (2009)
B5	Workflow configuration precedes P1/P2	Antecedent: workflow shapes engagement, control and delegation	McGuire et al. (2024)
B6	Perceived system agency moderates P5a	Moderator: system agency strengthens the agency-answerability link	Gray et al. (2007); Ciardo et al. (2020)

Analytic entries specify alignment or diagnosis. Empirical entries are conditional predictions; cited sources motivate or constrain them.

## 3 Generative AI and the decoupling of effort from product

Generative artificial intelligence presents a contemporary case of questions already raised by delegated and machine-assisted production. Photography challenged the relation between mechanical rendering and authorship: in Burrow-Giles Lithographic Co. v. Sarony (1884, pp. 59–60), the court emphasized the photographer’s intellectual conception and arrangement of the scene. Readymades, workshop and studio production, ghostwriting, and sampling likewise separate selection or direction from execution. Collective-production accounts make such distinctions intelligible (Becker, 1982). These precedents show why curation and direction can already furnish recognized grounds for authorship. They do not settle contemporary AI copyright rules or make every delegated contribution sufficient for every role.

The comparison can still be specified along three structures of creative production. The first is its causal structure: who or what brings the work about. A generative system can realize an initial text or image while the human specifies intent and selects among candidates, but human direction and revision can remain consequential. The second is its experiential structure: the predictive and retrospective cues through which a person experiences influence over the work. Delegation may reduce opportunities for continuous shaping, while control over goals, alternatives, and revisions can preserve other opportunities; research on automation and human-computer interaction motivates examining these possibilities (Berberian et al., 2012; Limerick et al., 2014). The third is its attribution structure: the grounds on which observers and the maker recognize contribution and answerability. Visible execution is one possible cue, alongside direction, verification, and authority over release. Changes across these structures need not occur together.

Several recent findings inform the account, but they concern different tasks and outcomes. Noy and Zhang (2023) document productivity gains from generative assistance in occupational writing tasks. Medeiros et al. (2025), studying word primes and divergent thinking, found no evidence of a general difference in Divergent Association Task scores by prime source or creativity level, except that a low-creativity prime presented as originating from ChatGPT yielded lower scores than other priming conditions. These findings constrain any general claim that generative assistance improves creative work. They do not jointly establish that accomplished output causes weaker ownership or observable disavowal.

Audience appraisal supplies a further distinction. Bellaiche et al. (2023) manipulated purported human versus AI origin while keeping AI-generated artworks constant; judgments depended on the source label, appraisal criterion, and perceived effort. These are audience evaluations, not creators’ ownership reports. Discussions of generative creativity identify ownership and credit as questions for changing creative practices (Rafner et al., 2023; Epstein et al., 2023). Sharma and Singhal (2026) review tensions between algorithmic optimization and human expression in creative industries. Those tensions motivate examination of authenticity standards, rather than a categorical claim that a painting based on an AI-suggested idea cannot be creative. That judgment requires considering the artist’s subsequent interpretation and production under specified criteria.

The decoupling makes the labor nexus visible in its contingency rather than creating a question that could not be posed earlier. A product can be attributed to a person even when much of its initial production is delegated; the designation, the person’s experience, and the contribution supporting the role may then diverge. P1 concerns the effort required for initial production: holding task and target quality comparable, greater delegation of externally specified production steps is predicted to reduce human effort at that stage. Setup and prompting remain part of that effort and can challenge the prediction if their costs outweigh the saving. Verification and revision costs are assessed separately, so total effort need not fall. The following sections examine when the resulting authorship role is adequately supported and when a deficit of legitimation arises. Neither designation nor responsibility automatically transfers to the machine.

## 4 Why a relational account of authorship is needed

The constructs psychology already possesses are powerful, and each addresses part of human–AI co-creation. Psychological ownership explains the feeling of mineness, agency research explains experienced influence, and responsibility research distinguishes several grounds of answerability and blame. The question developed here concerns their relation to a particular authorship designation. The same contribution and experience can support one role while leaving another unsupported. A relational account makes this comparison explicit and asks what follows when a designation exceeds its supporting grounds. It integrates the constituent concepts without requiring ownership, agency, and responsibility to weaken together or treating effort as their universal common cause.

Consider first psychological ownership. Its theory specifies the routes, control, intimate knowing, and investment of self, through which the feeling of mineness arises, and it has matured into a construct with a well-mapped nomological network (Pierce et al., 2001, 2003; Dawkins et al., 2017). Applied to co-creation, these routes make the character of engagement important: consequential prompting, selection, and revision may support ownership, whereas accepting an output with little involvement may offer fewer grounds for it. The ghostwriter effect, in which users report little ownership while declaring themselves authors (Draxler et al., 2024), shows why public attribution and felt ownership should be distinguished. Such a dissociation does not establish a deficit by itself. If an editorial role is supported by the person’s contribution and carries no requirement of strong felt ownership, low ownership need not undermine that role. The additional question is when a designation makes a claim that its experiential or contribution-based grounds cannot support, and whether that mismatch has consequences beyond low ownership alone.

Consider next the sense of agency. The experimental literature has concentrated on control over bodily movements and their immediate sensory effects, and on the cues that produce the feeling of authorship over such outcomes (Synofzik et al., 2008; Moore and Obhi, 2012; Haggard, 2017; Moore, 2016). Human–AI co-creation also involves distal decisions about what a work should express, which alternatives to select, and how to revise them. Agency over these decisions can differ from agency over the system’s detailed production. The cue-integration literature therefore supplies mechanisms for examining creative control, while measures developed for immediate action-effect episodes require task-specific justification before they are used to assess an extended work (Limerick et al., 2014). Ciardo et al. (2020) found that attributing intentional agency to a robot reduced participants’ own experienced agency in a joint task. This finding motivates a conditional hypothesis about generative workflows, rather than establishing the same effect in creative authorship. The relational account asks whether the influence a person experiences and exercises is sufficient for the role they claim. Limited agency over initial production can be compatible with a supported editorial role and with authority over later revision or release.

Consider the moral psychology of responsibility. Research distinguishes perceptions of AI moral agency and patiency (Ladak et al., 2024; Bonnefon et al., 2024), while aversion to machines making morally weighted decisions provides a further reason to examine judgments about artificial agents (Bigman and Gray, 2018). In philosophy of technology, a responsibility gap concerns difficulties assigning responsibility for the outputs of learning or autonomous systems (Matthias, 2004; Santoni de Sio and Mecacci, 2021). A mismatch in creative authorship need not produce such a gap. A person can feel little ownership of an output and still have a clear duty to check and stand behind its publication. Conversely, disagreement over creative credit can occur alongside agreement about who approved its release. Users’ felt answerability, observers’ judgments, and justified duties therefore require separate analysis. The framework relates authorship claims to their supporting grounds while preserving these different responsibility outcomes; it does not build diminished answerability into the diagnosis of a deficit.

Finally, the psychology of mind perception and anthropomorphism helps explain why a system can be interpreted as an intentional contributor. People attribute mind along the dimensions of agency and experience (Gray et al., 2007), and anthropomorphism has identifiable antecedents (Epley et al., 2007). Dennett's (1987) intentional stance concerns interpreting behavior through beliefs and desires. Applied to generative systems, this perspective can help explain a user’s causal interpretation of an output. Interpreting the system as a contributor does not establish that it can legitimately bear blame, nor that the user has lost ownership or responsibility. Mind perception thus offers a mechanism for experience and causal attribution, while the warrant for a human authorship role still depends on the relevant contribution and domain criteria. These literatures illuminate distinct relations. Bringing them together allows us to ask when a particular designation is supported, which ground is missing when it is not, and which psychological or social response might follow. That role-relative mismatch is the authorship legitimation deficit, to which we now turn.

## 5 The authorship legitimation deficit

The central claim of this article concerns a gap between an authorship designation and the grounds expected to support it. Delegating production can alter those grounds, but the implication depends on the role claimed and the standards applied. Because the term joins a familiar word to an unfamiliar use, the construct is worth defining slowly and distinguishing from the adjacent notions with which it is easily conflated.

An authorship legitimation deficit is a domain-relative mismatch in which a specific authorship designation exceeds the experiential or socially recognized contribution-based grounds expected to support that role under prior criteria. The construct turns on a family of distinctions that ordinary usage runs together. Designated authorship is the public assignment of a work to a person, by a byline, a signature, a platform record, or the person’s own declaration; it is a matter of who is named and in what role. Felt authorship concerns the first-person experience of the work as one’s own and of having shaped it; ownership and agency are assessed separately. Psychological ownership is the felt state of mineness, one component of that experience. Legal authorship allocates enforceable rights, while moral judgments of praise, blame, and answerability require their own grounds. Legitimated authorship, as used here, concerns a designation backed by the experiential or contribution-based warrant expected for the specified role. Effortful making can supply such grounds, but direction, selection, substantive editing, and other recognized contributions can also do so.

Consider a hypothetical personal-essay workshop whose brief, agreed before composition, distinguishes an editorial contributor from the author of a personally endorsed self-expressive essay. Both roles require substantive direction, selection, and revision, which the workshop editor assesses from drafts and an editing record. Only the second designation additionally asserts that the writer experiences the completed essay as their own work. This experiential requirement arises from the explicitly accepted brief, not from a general rule that communities may demand private feelings for ordinary authorship. Before selecting a designation, the writer reports whether that specified mineness claim fits the finished essay; the editor records the report but cannot infer it from the byline or contribution record. An affirmative report satisfies the experiential criterion, a negative report identifies a shortfall, and a missing or equivocal report leaves it undetermined. Suppose the writer directs, selects, and substantively revises generated passages, experiences agency over those changes, but reports that the essay does not feel like their own. Contribution-based warrant is adequate and agency is intact, while the self-expressive designation has an experiential shortfall. The same record supports editorial credit without requiring mineness. This constructed example illustrates an explicitly agreed role criterion, rather than an established workshop norm. Low ownership otherwise supplies no experiential deficit, and strong ownership cannot remedy insufficient contribution-based warrant.

Three features of the construct deserve emphasis. First, it is a condition at the interface of individual experience and social-normative structure, not a mere feeling. Diagnosis identifies the person, work, authorship role, domain, and evaluator, together with the criteria applied to that role. An experiential expectation is diagnostic only when it forms part of the specified role claim and is accepted in advance; an audience’s preference for an author to feel ownership does not by itself create that requirement. Ownership is assessed against that expectation, agency against expected influence, and community warrant against criteria for recognizing contributions. A facet applies only where the role has a corresponding requirement. The criteria are established independently of the focal person’s later decision to accept, reject, or qualify the designation. This separates the grounds of diagnosis from the behavioral consequences discussed below.

Second, the deficit is a multidimensional relational condition whose facets need not vary together. A person may have adequate agency and contribution-based warrant but weak ownership, or strong ownership with inadequate warrant for the role claimed. A deficit is identified where an applicable ground falls short of its prior criterion; the degree of shortfall can then be examined within that facet. Profiles can be ordered when their applicable facets consistently indicate greater or lesser mismatch under the same criteria. Opposing differences remain distinct profiles rather than being forced into a single severity ranking. A reflective factor or formative index would require an additional justification of covariance or weights. Nor is the deficit obtained by subtracting a binary designation from an ownership score: difference-score models impose assumptions and restrictions on their components (Edwards, 2001). Separate measures and role-relative profiles provide a starting point for examining the proposed relation.

Third, diagnosis under a community’s standards is distinct from endorsing those standards. An audience’s recognition describes a social judgment; whether its criteria fairly and adequately credit contributions requires a further normative argument. A deficit under prevailing criteria does not by itself show wrongdoing or lack of worth in the work. Likewise, legal rights and justified responsibility remain separate questions. The term legitimation here concerns the grounds supporting an authorship role. A recognized role can rest on unjust criteria, an unsupported claim can arise without deception, and release duties can persist despite little felt ownership.

Figure 2 summarizes the proposed model and links its relations to the propositions developed below. The labor nexus organizes the routes through which engagement, control, and contribution information can support experiences and judgments. P2d describes their possible alignment, and P3 identifies a role-ground mismatch as a deficit; these are analytic relations, not additional empirical effects. P4 and P6 propose conditional responses to diagnosed mismatches. P5a and P5b concern agency and audience evaluation through their existing literatures; they are not consequences of the deficit. These responses are assessed independently rather than being used to establish the deficit by definition. Each empirical arrow has a numbered proposition, while diagnostic relations and contextual conditions are distinguished in the figure and in Table 2.

**Figure 2:**
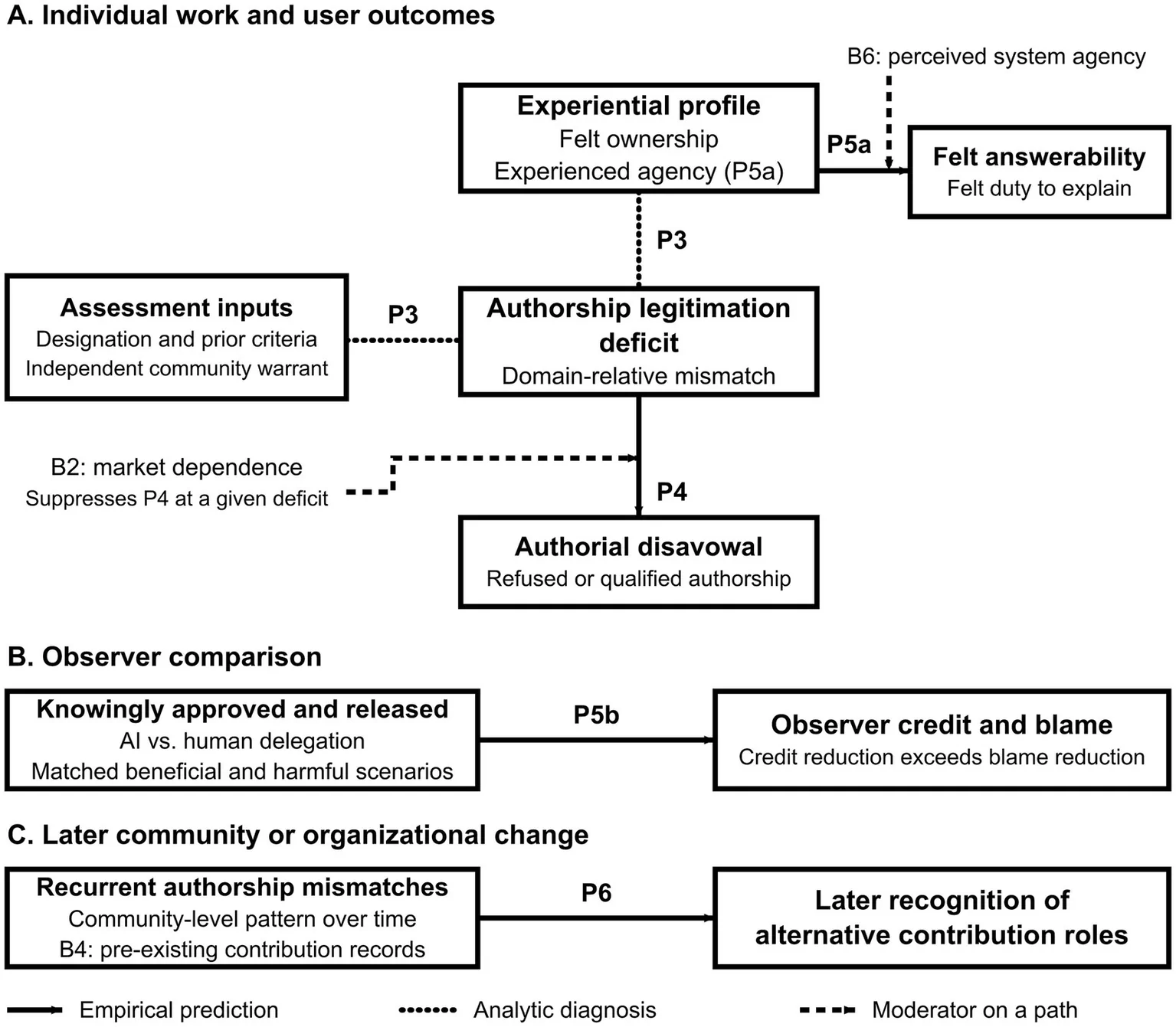
Analytic diagnosis and distinct outcome domains. Solid arrows denote conditional empirical predictions; dotted connectors without arrowheads denote analytic diagnosis (P3); dashed arrows denote moderators. **(A)** Prior designation and domain criteria, independently assessed contribution-based warrant, and applicable experiential expectations inform diagnosis. Responsibility is an outcome, not a diagnostic component. P4 and P6 use the relational diagnosis; P5a and P5b are component and comparative hypotheses. P4 concerns later refusal or qualification among users who accept the prior criteria and have low-cost alternatives. B2 predicts suppression when qualification threatens income. P5a originates only from the experienced-agency facet, not an ownership-agency composite or the deficit. Perceived system agency (B6) strengthens the agency-felt-answerability association under fixed release duties. **(B)** P5b compares observer credit and blame for matched AI and human delegation, holding the approving person’s contribution, knowledge, release authority, and consequences comparable. It predicts a larger credit reduction than blame reduction, not a deficit-induced increase in blame. **(C)** P6 conditionally relates recurrent community-level mismatches to later recognition of alternative contributions; B4 concerns records available before that change. Tables 2, 4 specify P1/P2 and workflow configuration (B5).

## 6 Propositions and mechanisms

This section states the propositions that constitute the model and analyzes its three outcome domains in turn. The propositions are collected in Table 2, including the decomposition of the labor nexus set out in Section 2; the outcomes are summarized in Table 3. P1 predicts a reduction in initial-production effort with greater delegation of production steps, under comparable tasks and target quality. P2 summarizes the workflow-specific routes to ownership and agency in P2a and P2b, while P2c separately concerns observers’ answerability judgments. P2d describes possible alignment among these relations. P3 is the analytic diagnosis of a mismatch between a designation and its applicable grounds, as defined in Section 5; it is not another causal effect of weakened ownership. P4 uses the role-ground relation to predict later refusal or qualification, and P6 extends that relation to subsequent community recognition. P5a concerns experienced agency and P5b concerns observers’ credit and blame; neither depends on a diagnosed deficit. Each outcome is assessed independently, with its own mechanism, scope conditions, and disconfirmation criterion.

**Table 3:** Outcome domains and conditional predictions in the authorship model.

Proposed outcome	Definition	Mechanism	Observable manifestation	Role in the framework
Authorial disavowal (P4)	Refusal or qualification of a designated authorship claim	A role-ground mismatch challenges a claim the person understands and accepts	Qualifying unsupported authorship or choosing a supported contribution label	Relational hypothesis about a later response to a diagnosed mismatch
Felt answerability (P5a)	The user’s felt obligation to explain and stand behind release	Reduced agency and perceived system contribution may weaken felt personal implication	Lower reported obligation under matched release authority and duties	Component hypothesis about agency; a deficit diagnosis is not required
Observer credit and blame (P5b)	Observers’ credit for beneficial outputs and blame for harmful outputs under matched AI and human delegation	Credit can depend on how audiences value human or AI generation; blame can rest on knowing approval and release	Smaller reduction in blame than credit when an AI delegate replaces a human delegate; comparable contrasts	Comparative hypothesis about audience evaluation; a deficit diagnosis is not required
Reorganization of legitimation (P6)	Later community recognition of alternative contribution grounds	Recurring mismatches and assessable records facilitate coordination over narrower roles	Later uptake of direction, selection, editing or verification as recognized role grounds	Community-level relational extension; norm change remains conditional

### 6.1 Authorial disavowal

Authorial disavowal is the observable refusal or qualification of an offered authorship designation. Its proposed mechanism is a collision between a claim and the grounds the person understands and accepts as required for that claim. A person may regard a work as accomplished yet judge that a sole-creator designation overstates their contribution. Where a narrower label is available, qualification can resolve that mismatch without withholding the work itself. Low felt ownership, concealment of AI use, and continued unqualified claiming are distinct observations; none is relabeled as hidden disavowal. Draxler et al. (2024) document continued authorship declarations despite weak ownership, which limits any inference from non-ownership to refusal. Social penalties for AI use provide a competing explanation for concealment (Reif et al., 2025), rather than evidence for the proposed mechanism.

P4 predicts greater refusal or qualification at a given output quality when the independently assessed role-ground mismatch is greater, among people with accurate contribution information who understand and accept the relevant criteria and have low-cost alternatives. The designation, contribution information, and prior criteria are recorded before the authorship choice, together with the person’s understanding and acceptance of those criteria. In the workshop example, writers whose contributions support both roles but who report the ownership shortfall should more often decline or qualify the self-expressive designation than the supported editorial role. They can accept editorial credit or revise the essay before reconsidering the stronger claim. Ownership and agency are recorded before this choice and held comparable across the offered roles; qualification is a separate outcome. Equal or lower qualification of the unsupported designation would challenge P4 within its stated scope. Where presenting the work without qualification is necessary for income, observable disavowal may be suppressed; B2 specifies this cost-dependent extension. Continued claiming in that condition is recorded as continued claiming, not counted as confirmation of P4.

### 6.2 Felt answerability and observers’ responsibility judgments

Responsibility displacement concerns a possible change in the user’s felt locus of answerability, not a loss of assigned duties. A person who experiences little influence over a work’s content may interpret the system as its source and feel less obligation to explain the creative choices embodied in it. Mind perception makes that causal interpretation available (Gray et al., 2007; Dennett, 1987). Ciardo et al. (2020) found reduced experienced agency when participants attributed intentional agency to a robot in a joint task; extension to creative work remains a hypothesis. The mechanism concerns the agency facet of the authorship relation. Ownership, justified responsibility, and the audience’s allocation of blame are assessed separately.

P5a predicts a positive association between users’ experienced agency over creative decisions and felt answerability for the output, holding knowledge, approval or release authority, and assigned duties constant. Perceived system agency (B6) is expected to strengthen that association: when the system is seen as an intentional contributor, a user’s limited experienced influence provides a more salient alternative causal source, whereas substantial experienced influence supports the user’s own answerability. This conditional prediction neither excuses a publisher from checking an output nor requires responsibility to fall whenever ownership is weak. A null or reversed association within the fixed-authority scope, or no strengthening with perceived system agency, would challenge the corresponding prediction.

Observers can follow a different route. They can recognize limited creative contribution while continuing to hold the person answerable for approving or releasing a harmful output. Porsdam Mann et al. (2023) develop a conceptual argument about credit-blame asymmetry; it supplies a comparison to examine rather than an established general effect. Driving studies further constrain the original displacement claim: Awad et al. (2020) found that humans were blamed more than automated cars when both made mistakes, and Beckers et al. (2022) found human blame for crashes drivers could not reasonably avoid. These findings do not establish creative-authorship effects, but they show why reduced agency cannot be assumed to move observer blame away from humans.

P5b therefore tests a separate, pre-specified comparison: for the same human approval or release role, contribution, and knowledge, replacing a human delegate with an AI delegate is predicted to reduce creative credit for beneficial outputs more than blame for harmful outputs. The beneficial and harmful reference scenarios are fixed, with outcome magnitude and foreseeable consequences matched across delegate conditions. Credit and blame are measured separately using comparable response scales and fixed reference scenarios; they are not subtracted from unlike measures of ownership or designation. Equal or greater reduction in blame would challenge the asymmetry. The comparison isolates the source of delegated creation while keeping the focal person’s contribution and release decisions fixed. Any asymmetry is a proposed audience response to that source, rather than evidence that the focal person’s contribution changed. It is not an arrow from overall deficit severity to uniformly increased or decreased blame, and it does not require P4 or P5a to hold.

### 6.3 Reorganization of legitimation

A third consequence concerns the community rather than only the individual creator. Producing more and better work with less effort can improve a person’s position while leaving a particular authorship claim unsupported under existing criteria. Communities encountering recurrent mismatches may seek alternative grounds for recognition. Reorganization of legitimation is the later change in which contributions a community accepts as supporting specified roles. Candidate grounds include direction, interpretive selection, substantive editing, verification, and accountable publication. These grounds have historical precedents; their contemporary uptake need not represent an entirely new form of authorship.

The proposed mechanism is substitution under constraint. When a familiar designation no longer communicates who directed, shaped, or checked a work, more explicit contribution roles can make claims easier to assess. Which roles gain recognition depends on whether relevant audiences accept the documented contributions, in the way that valuation depends on social arrangements that establish worth (Beckert, 2009; Aspers, 2018; Elder-Vass, 2018). Debates over ownership and credit in generative creativity make this question salient (Epstein et al., 2023). P6 predicts greater subsequent recognition of alternative contribution grounds in otherwise comparable communities encountering more recurrent, independently documented mismatches, especially where pre-existing contribution records make those grounds assessable (B4). Baseline records and later recognition are separate observations. Persistent prior criteria, exclusion, or disclosure penalties remain competing responses; unchanged or reduced recognition under the stated conditions would challenge P6. Temporal ordering alone would not establish causality.

An editing history illustrates the distinction. A record can show which passages a person substantially revised and who verified and approved release. An editor or client can then assess an editorial claim without assuming sole creation of the initial material. Recognition of that narrower role can improve contribution-based warrant while leaving the person’s felt ownership unchanged. Conversely, a disclosure stating only that AI was used neither shows the human contribution nor guarantees recognition. Infrastructure can make relevant work legible; it cannot supply a contribution that did not occur or ensure that an audience values it.

## 7 Boundary conditions and moderators

The authorship legitimation deficit is not uniform across fields or situations. This section specifies six contextual conditions, distinguishing prior role criteria (B1 and B3), an empirical moderator of authorship choices (B2), baseline contribution records (B4), workflow configuration (B5), and perceived system agency (B6). Each is assessed independently of the focal outcome. Table 4 identifies the relation concerned and the direction proposed; not every condition is a moderator of deficit severity.

**Table 4:** Boundary conditions of the authorship legitimation deficit.

Context or workflow condition	Relevant relation	Direction or diagnostic role	Implication for future research
Visibility of effort (B1)	Prior effort criterion informing P3	Low-execution claims have less warrant where visible effort is highly valued; weaker criteria permit other grounds	Elicit effort criteria independently; compare warranted roles at matched contribution
Market dependence (B2)	Financial-cost condition on P4	At a given deficit, income-threatening qualification suppresses observable refusal; no threat predicts no economic suppression	Measure income dependence and qualification cost separately from deficit
Originality-norm strength (B3)	Prior origination criterion informing P3	Singular-origination criteria yield less warrant for delegated roles; distributed-role criteria can support curation or direction	Compare prior role criteria and contribution-based warrant across domains
Attribution infrastructure (B4)	Pre-existing contribution records conditioning P6	Editing histories, disclosures and named release authority make contributions assessable; informative records allow evaluation without guaranteeing recognition	Record baseline infrastructure and later recognition separately; assess exclusion and disclosure penalties
Workflow configuration (B5)	Workflow antecedent to P1/P2	Direction, iterative shaping, substantive editing and selection offer different engagement/control opportunities; execution effort is a separate variable	Vary workflow role and revision timing; measure effort, ownership and agency separately
Perceived system agency (B6)	Empirical moderator of P5a	Greater attributed system agency strengthens the agency-answerability association under fixed release authority and duties	Assess perceived system agency separately; test its interaction under matched duties

The first condition is the visibility and legibility of effort in a field (B1). It concerns a community’s prior use of visible production effort as a criterion of creative credit. Where effort is conspicuous and treated as a mark of worth, an otherwise comparable low-production-effort contribution may receive weaker warrant for a sole-creator designation. Where effort is obscured or discounted, other grounds may carry more weight. The effort heuristic motivates examining this condition but concerns evaluations of quality and value, especially under ambiguity, rather than authorship warrant itself (Kruger et al., 2004). B1 is assessed through prior evaluative rules or independent audience judgments before the focal claim is evaluated. Actual individual effort and the resulting warrant remain separate measures.

The second condition is market dependence (B2), which moderates the path from deficit to observable authorial disavowal (P4). Where people depend on income from presenting the work without qualification, refusal or qualification is costly. At a given independently assessed mismatch, greater income dependence is therefore predicted to suppress observable disavowal when qualification threatens income. Discomfort may persist, but it is not the behavioral outcome. Income dependence and the anticipated cost of qualification are assessed separately. Where qualification carries no income threat, this economic mechanism predicts no suppression.

The third condition is the strength of the originality norm in the field (B3). Where a specified role requires singular personal origination, delegated production may provide less recognized warrant for it. Where authorship is understood as coordination of a distributed process (Becker, 1982), direction, selection, or substantive editing may support a legitimate role without extensive physical execution. B3 concerns which kind of contribution is accepted; B1 concerns the emphasis placed on visible effort. They may covary and are measured separately rather than assumed to be statistically independent. Contrasts between solo-origin and collective-authorship domains must establish the criteria before judging a focal work, rather than infer the norm from a person’s later refusal.

The fourth condition is attribution and accountability infrastructure already present before the change studied under P6 (B4). Such arrangements include a contribution statement distinguishing drafting, direction, selection, and editing; an editing history identifying substantive revisions; disclosure of the generative system used; and a named person responsible for verification and release. These records allow a client or editor to evaluate what a person actually did and to assign a narrower role when warranted. They can reduce uncertainty about contribution without restoring felt ownership. Their informational effect depends on recording identifiable contributions in a form audiences can examine; subsequent acceptance of new contribution grounds is the outcome under P6. Disclosure alone may attract social penalties (Reif et al., 2025), and a platform attribution label does not verify a claim. B4 records baseline availability and quality of information; P6 measures later changes in recognized grounds. Treating them as separate stages avoids defining infrastructure by the recognition it is supposed to explain.

The fifth condition is active human involvement, specified as workflow configuration (B5). It distinguishes setting the initial direction, iteratively shaping content, editing a completed draft, and selecting among candidates. Configuration affects the amount and kind of delegated production in P1 and the opportunities for control, knowledge, and self-investment in P2. It is not defined as simply the inverse of low effort: high discretion can coexist with little execution, and extensive checking can coexist with little creative direction. McGuire et al. (2024) compared substantive editing with iterative poetry co-creation and measured creativity and creative self-efficacy. Their findings do not demonstrate ownership recovery. The actionable hypothesis is that consequential shaping can support ownership and agency; its strength depends on the task, timing, and discretion afforded, and requires separate outcome measures.

The sixth condition is perceived system agency (B6), the user’s attribution of intentional contribution to the system. It is assessed separately from the user’s experienced agency and felt answerability. Mind-perception research and Ciardo et al.'s (2020) joint-task finding motivate this condition (Gray et al., 2007), without establishing its effect in creative authorship. Under P5a’s fixed-authority scope, higher perceived system agency is predicted to strengthen the positive association between experienced human agency and felt answerability. Observer credit and blame remain the separate P5b comparison.

## 8 Implications for theory and future research

The account has implications for several strands of psychological theory and a corresponding set of testable predictions. For theoretical and philosophical psychology, it clarifies a relation among familiar concepts. Authorship can be supported by felt ownership, experienced influence, and recognized contributions, while answerability also depends on knowledge and authority. The framework asks whether those grounds support a particular role. Its incremental claim is that a role-ground mismatch provides information beyond any one component: the same ownership and contribution profile can warrant editorial credit but fail to warrant sole creation. This comparison separates the relational claim from a general label for low ownership. The main contribution is this role-relative classification. Its empirical extension asks whether the patterned fit between roles and grounds predicts outcomes beyond their separate main effects, rather than positing an additional causal entity independent of those components. P4 and P6 test that extension; P5a and P5b do not. If relational comparisons add no predictive value, the classification can still clarify supported and unsupported claims without supplying an additional explanation of responsibility.

For the constituent theories, the account identifies connected questions. Psychological ownership research can examine how people manage a divergence between public designation and felt ownership (Pierce et al., 2003; Dawkins et al., 2017; Draxler et al., 2024), including when it is tolerated because a contribution role is adequately supported. Agency research can distinguish influence over detailed production from distal control over direction, selection, and revision (Synofzik et al., 2008; Limerick et al., 2014). Intentional binding concerns a defined action-effect episode; assessing authorship of an extended work requires justification of task-specific measures. Responsibility research can test why a person’s felt answerability and an observer’s blame judgment diverge when creative influence is limited but release authority persists (Bonnefon et al., 2024). These are extensions and comparisons grounded in the existing concepts, rather than claims that those literatures cannot address co-creation.

For human–AI co-creation and its design, interventions aimed at productivity address a different outcome from supported authorship (Rafner et al., 2023). Two directions follow. Consequential human shaping may support ownership and agency, whereas clearer contribution roles and records can make warranted claims assessable without necessarily changing experience. Testing these predictions requires recording the designation, documented contribution, evaluator, and prior criteria before observing acceptance, refusal, or qualification. Ownership items can be adapted to the focal work with validity assessed in that setting (Van Dyne and Pierce, 2004); experienced influence, opportunities for consequential revision, and community recognition require separate measures. Criteria should not be inferred from the very authorship choice the model predicts. Independent domain members assess whether documented contributions support the offered role under the prior criteria and state their reasons before seeing the person’s authorship choice. Their judgments, the person’s acceptance of the criteria, and disagreements among audiences are recorded separately.

A creative-task comparison can vary delegated production steps and shaping opportunities separately while holding task and target quality comparable. This examines initial-production effort (P1) and ownership and agency routes (P2a/P2b), rather than treating an editor label as a proxy for passivity. P4 requires crossing designated roles with documented contribution profiles under prior criteria, so that the same label is supported in one profile and unsupported in another. Qualification should track the unsupported combinations beyond main effects of contribution, ownership, agency, role labels, and anticipated stigma. A separate qualification-cost comparison examines B2. Fixed-authority scenarios can examine users’ felt answerability and perceived system agency (P5a/B6), while matched AI-versus-human delegation scenarios assess observer credit and blame independently (P5b). Longitudinal community comparisons are needed for P6, with baseline records, recurring mismatches, prior norms, and later recognition recorded separately. Rival explanations include source-label penalties, institutional incentives, and unchanged hierarchies. Facet and profile comparisons retain their distinct measurement assumptions rather than defaulting to a single deficit scale (Edwards, 2001).

## 9 Discussion

The model’s limits concern its explanatory reach and the measures needed to assess it. Existing findings establish task-specific relations among output evaluation, ownership, creative self-efficacy, and agency; they do not jointly validate the proposed deficit or its consequences. A well-evaluated work can be appropriately credited, weakly owned, and fully accountable. Separating these relations allows different responses: changes in workflow may affect experience, accurate contribution roles may affect recognition, and verification duties may sustain answerability. The classification specifies which grounds apply to a role. Additional explanatory reach rests on P4 and P6 surviving tests against separate component effects and stigma-based alternatives; the responsibility hypotheses remain grounded in agency and audience evaluation.

Two further boundaries deserve note. First, the standards determining supported authorship are not universal. Conceptions of ownership, agency, and the self vary across cultures, while originality norms and the visibility of effort differ across fields and traditions. Diagnosis therefore specifies the relevant role, evaluator, and criteria; disagreement among audiences is retained rather than collapsed into a single normative baseline. Recognition under a community’s standards does not establish that those standards are fair. Second, a deficit may be transitional. Communities can narrow a designation, recognize different contributions, or revise workflows, while others may preserve unequal credit or intensify disclosure penalties (Elder-Vass, 2018; Beckert, 2009; Reif et al., 2025). The model does not predict inevitable reorganization or persistent disavowal in every unresolved case. P4 and P6 use diagnosed mismatches under specified conditions; P5a and P5b test component and comparative mechanisms.

The contribution, finally, is a matter of reframing as well as of construction. A phenomenon also examined through ethics, law, and the sociology of technology is posed here as a psychological and relational problem: how a person experiences a product as their own, feels like its agent, and answers for its release, and how those relations support the role assigned to that person. Historical delegation and contemporary generative workflows both make this comparison necessary. It connects psychological ownership, the sense of agency, and responsibility research while preserving their different outcomes, and returns a sharpened set of questions about when an authorship claim is adequately supported.

## 10 Conclusion

Authorship can align feeling that a work is one’s own, feeling like its agent, and standing answerable for it. Effortful making can support those relations, an arrangement this article calls the labor nexus of authorship, while direction, selection, editing, and authority over release provide other grounds. Generative workflows can separate initial-production effort from product without uniformly removing those grounds. An authorship legitimation deficit arises when a specified role exceeds its applicable experiential or contribution-based support under prior domain criteria. The contribution is a role-relative conceptual classification. P4 and P6 propose conditional responses to the identified mismatch; P5a and P5b formulate related hypotheses about agency and audience evaluation, rather than deficit-based responsibility predictions. The same limited production effort can support editorial credit while leaving a sole-creator claim unsupported, and low felt ownership can coexist with full responsibility for release. Understanding these differences focuses the psychology of authorship on the relation between a designation and its grounds rather than on productivity or effort alone.
